# General Adaptation in Critical Illness: Glucocorticoid Receptor-alpha Master Regulator of Homeostatic Corrections

**DOI:** 10.3389/fendo.2020.00161

**Published:** 2020-04-22

**Authors:** Gianfranco Umberto Meduri, George P. Chrousos

**Affiliations:** ^1^Division of Pulmonary, Critical Care, and Sleep Medicine, Department of Medicine, University of Tennessee Health Science Center, Memphis, TN, United States; ^2^Memphis Veterans Affairs Medical Center, Memphis, TN, United States; ^3^University Research Institute of Maternal and Child Health and Precision Medicine, National and Kapodistrian University of Athens, Medical School, Athens, Greece

**Keywords:** critical illness, glucocorticoid receptor-alpha, nuclear factor-κB, mitochondria, hypovitaminosis

## Abstract

In critical illness, homeostatic corrections representing the culmination of hundreds of millions of years of evolution, are modulated by the activated glucocorticoid receptor alpha (GRα) and are associated with an enormous bioenergetic and metabolic cost. Appreciation of how homeostatic corrections work and how they evolved provides a conceptual framework to understand the complex pathobiology of critical illness. Emerging literature place the activated GRα at the center of all phases of disease development and resolution, including activation and re-enforcement of innate immunity, downregulation of pro-inflammatory transcription factors, and restoration of anatomy and function. By the time critically ill patients necessitate vital organ support for survival, they have reached near exhaustion or exhaustion of neuroendocrine homeostatic compensation, cell bio-energetic and adaptation functions, and reserves of vital micronutrients. We review how critical illness-related corticosteroid insufficiency, mitochondrial dysfunction/damage, and hypovitaminosis collectively interact to accelerate an anti-homeostatic active process of natural selection. Importantly, the allostatic overload imposed by these homeostatic corrections impacts negatively on both acute and long-term morbidity and mortality. Since the bioenergetic and metabolic reserves to support homeostatic corrections are time-limited, early interventions should be directed at increasing GRα and mitochondria number and function. Present understanding of the activated GC-GRα's role in immunomodulation and disease resolution should be taken into account when re-evaluating how to administer glucocorticoid treatment and co-interventions to improve cellular responsiveness. The activated GRα interdependence with functional mitochondria and three vitamin reserves (B1, C, and D) provides a rationale for co-interventions that include prolonged glucocorticoid treatment in association with rapid correction of hypovitaminosis.

## Critical Illness, Species Evolution, and Individual Development

The reasons behind the evolutionary success of mammals and other multicellular organisms is their extraordinary capacity to adapt to changing environmental conditions and survive by maintaining their homeostasis (1). Homeostasis refers to the relative stability in the activity of the physiological systems of the organism that are essential to support life (2). The process of maintaining stability within a harmless range via homeostatic physiologic corrections to both predictable and unpredictable adverse forces or stressors is termed “eustasis” (2). In the course of human evolution, homeostatic corrections have emerged to increase the host's ability to cope with adverse or even catastrophic events (3). These responses are shaped by trade-offs, sometimes with benefits and disadvantages in different periods of the life cycle (4). Following the Cambrian explosion, about 450 million years ago, when multicellular organisms—originally formed in water environments—colonized the land physiological homeostatic changes emerged to allow survival. These corrective changes were essential to mammalian species evolution and emerged to solve a frequent conflict between environmental changes and preservation of the individual allowing survival and, hence, reproduction. These alterations permitted progression to future generations i.e., survival of the species through the active process of natural selection (5). These corrections involved profound neural, metabolic and immune changes mediated by a few major physiological systems (e.g., the central nervous, autonomic, cardiorespiratory, endocrine, and immune systems), and acting through integrated crosstalk pathways, that was associated with appropriate responses throughout the organism (5). Such changes have been relatively conserved across many vertebrates, including mammalian species (6, 7). They have evolved to allow coping with lack of energy, dehydration, hemorrhage, infections, toxic substances, or relatively short-lived inflammatory responses, such as those of wound healing or exposure to foreign substances (7).

When the organism is exposed to stressors that exceed the harmless stability range, individual survival is maintained at the expense of this organism's health and longevity. This condition is different from healthy homeostasis or eustasis and is called “allo-stasis” or different (allo)-stasis or even more accurately “caco (bad) –stasis. The cumulative cost of cacostasis for the organism, has been called allostatic or cacostatic burden. Excessive or prolonged cacostatic burden results in severe acute and/or chronic cacostatic pathology (2, 8). The intensive care unit (ICU) stress state represents a new and very different ecosystem from those within which humans evolved in the past. Actually, critical illness epitomizes an acute and/or chronic cacostatic burden that goes beyond an evolutionarily conserved physiological adaptive response, and if left untreated it could rapidly exhaust homeostatic compensation and lead to death of an organism (lethal cacostasis). In critically ill patients, the need for vital organ support (maintenance of arterial blood pressure, mechanical ventilation, and other support measures, which were not available until the middle of the last century), reflects near exhaustion or exhaustion of (i) neuroendocrine homeostatic compensation, also known as “critical illness-related corticosteroid insufficiency” (CIRCI) (8); (ii) cell bio-energetic and other functions; and (iii) reserves of vital micronutrients (vitamins and minerals). Even when it allows survival of the patient, homeostatic failure, ranging in acuity and severity, has a major impact on morbidity and mortality during and after hospitalization. CIRCI-associated dysregulated systemic inflammation and mitochondrial dysfunction are central to the increased morbidity and mortality of acute and/or chronic critical illness and the subject of this review.

## Innate Immunity and Nuclear Factor-κB

In multicellular organisms, the innate immune system with its recognition and signaling mechanisms is the most ancient form of host defense to infectious and non-infectious threats during evolution (9). The nuclear factor-κB (NF-κB) system, a “rapidly-acting” primary transcription factor regulated cellular response, is a central activator of innate immunity. It appears that the NF-κB was incorporated into this ancient signaling pathway more than 420 million years ago, and has been shown to play independent roles in vertebrate and insect lineages (9, 10). In most cell types, the inactive form of NF-κB, a heterodimeric protein composed of the DNA-binding proteins p65 and p50, is retained in the cytosol by association with inhibitory factors, such as IκB proteins; when activated, the latter are rapidly phosphorylated and degraded via the proteasomal pathway, liberating NF-κB and allowing it to translocate into the nucleus (11). In tumor necrosis factor-α (TNF-α)-stimulated HeLa cells, a genome-wide study identified 12,552 genome binding sites of p65 (12). Among the 1,667 distinct NF-κB target genes identified, NF-κB controlled the expression of 249 target genes, including inflammatory cytokines, chemokines, inflammatory enzymes, adhesion molecules, and immune system receptors, which are known to orchestrate the integrated inflammatory and immune responses. Interestingly, an additional 626 identified NF-κB target genes were involved in metabolic processes (13).

**Figure 1:**
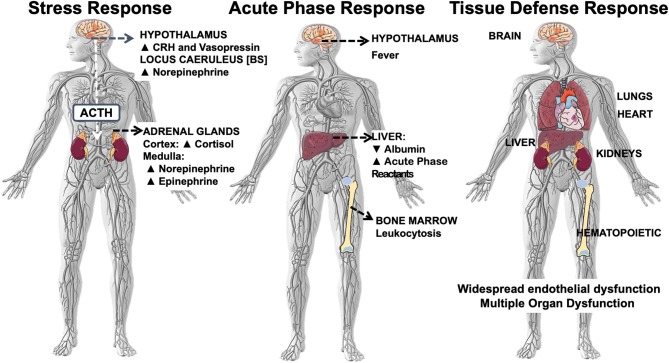
Systemic inflammation-associated multi-system responses: stress, acute phase, tissue defense. Systemic inflammation-associated multi-system response that includes at least three major domains: (i) the stress system composed by the hypothalamic-pituitary-adrenal (HPA) axis and the locus caeruleus-norepinephrine/sympathetic nervous system; (ii) the acute-phase reaction (APR), and (iii) the tissue defense response of the target organs.

In critical illness, NF-κB-driven systemic inflammation, also known as a “*cytokine storm*” (14), activates a multi-system response that includes at least three major domains: (i) the stress system composed by the hypothalamic-pituitary-adrenal (HPA) axis and the locus caeruleus-norepinephrine/sympathetic nervous system activated to provide sufficient energy and hemodynamic stability to overcome the initial phase of critical illness (15); (ii) the acute-phase reaction (APR), which has several adaptive functions, including increasing the production of procoagulant factors in preparation for possible tissue damage (16); and (iii) the tissue defense response (TDR) of the target organs [Figure 1; (11, 17)]. The main effectors of systemic inflammation are the inflammatory cytokines, the acute-phase reactants, and the peripheral effectors of the sensory afferent nervous system. The inflammatory cytokines include TNF-α, interleukin-1β (IL-1β), IL-6, chemokines, and other mediators of inflammation. The acute-phase reactants are mostly of hepatic origin and include the C-reactive protein (CRP), fibrinogen, and plasminogen activator inhibitor-1. During the acute-phase reaction, myelopoiesis is favored at the expense of both lymphopoiesis and hematopoiesis, explaining in part the persistent lymphopenia and anemia of critical illness (18). Substance P is an example of an effector of the sensory afferent nervous system, while hypothalamic corticotropin releasing hormone (CRH), vasopressin, cortisol, the catecholamines (norepinephrine and epinephrine), and peripheral neuronal CRH represent effectors of the stress system [reviewed in (19)].

The tissue defense response is an integrated network of three simultaneously NF-κB-activated pathways that account for much of the histological, physiological, and laboratory changes observed in vital organs during critical illness (11). These three pathways are those of tissue inflammation, hemostasis, and tissue damage repair: (i) tissue inflammation includes changes in the endothelium, such as loss of the glycocalyx, adhesion/diapedesis of activated neutrophils, endothelial injury, increased porosity with interstitial exudative edema, and loss of vascular tone, and changes in the epithelium, such as loss of integrity and cell apoptosis; (ii) the hemostasis pathway includes platelet activation and aggregation, intravascular clotting with decreased microvascular patency, extra-vascular fibrin deposition, and, lastly, inhibition of fibrinolysis, and (iii) tissue damage repair includes regenerating native parenchymal cells, fibroproliferation and deposition of extracellular matrix, resolution of granulation tissue, and clearance of apoptotic cells and debris (11).

The roles of macrophages and mitochondria in homeostatic corrections is the subject of intense research. Mononuclear phagocytic cells (MPCs), including macrophages and dendritic cells, are widely distributed throughout the tissues of the organism, where they perform essential homeostatic, surveillance, and regenerative tasks. As the neuro-endocrine-immune response progresses, macrophages change phenotype and play an essential role in both innate and adaptive immune responses, in the resolution of inflammation and in the tissue repair and regeneration (discussed further in section Glucocorticoid Receptor-Alpha and Homeostatic Corrections) (20). Mitochondria are targets of GR and critical modulators of homeostatic corrections owing to their critical role in energy production, synthesis of stress-associated steroid hormones, and their capacity to generate signals that promote cellular adaptation (see section Cellular Energetics—Mitochondrial Function) (21).

Systemic homeostatic corrections are driven by elevated levels of circulating inflammatory cytokines, and based on disease progression, can be broadly divided into either adaptive/resolving (regulated systemic inflammation) vs. maladaptive/unresolving (dysregulated systemic inflammation) (11, 14). Evidence from the literature on severe sepsis (22–30), acute respiratory distress syndrome (ARDS) (31–35), and trauma (14, 36) provides strong support that the degree of NF-κB-driven elevation in inflammatory biomarkers at ICU entry and during ICU stay correlates with disease severity and hospital mortality (33, 37–39). In addition to elevated inflammatory markers, critically ill patients have profound activation of the coagulation system (elevated D-dimer levels, prolonged prothrombin time and activated partial thromboplastin time, and reduced levels of the anticoagulant proteins, protein C and antithrombin) and evidence of endothelial cell activation with disturbed vascular integrity that correlates with disease severity and outcome (see section Endothelium) (40–42). Evidence that hemostasis and inflammation evolved from a single-triggered mechanism can be traced back more than 450 million years ago, based on studies with the horse-shoe crab (*Limulus polyphemus*) (43).

In hospital survivors, failure to achieve homeostatic correction has a significant negative long-term impact, with experimental work suggesting that it might potentiate the peripheral and brain pro-inflammatory cytokine response to a subsequent inflammatory challenge (44). Independently of age and comorbidities, patients with elevated circulating biomarkers of inflammation and hemostasis at hospital discharge have persistent elevation over time with increased risk for cardiovascular events, re-hospitalizations, and 1-year mortality (41, 45, 46). “*Persistent Inflammation, Immunosuppression, and Catabolism Syndrome*” (PICS) has been postulated as the underlying pathophysiology of chronic critical illness (CCI) (18, 47). About 50% of sepsis patients have a debilitating condition characterized by a self-perpetuating cycle of persistent low-grade systemic inflammation mimicking chronic stress (elevated cortisol) (44, 48, 49), glucocorticoid resistance, altered hemostasis (50, 51), mitochondrial dysfunction (52, 53), and accelerated inflamm-aging (9, 54), with increased risk for chronic inflammatory systemic diseases (7, 55). The evolutionary trade-off between acutely beneficial and chronically harmful homeostatic corrections was the subject of a recent review (7).

Recently, a critical role was identified for the *FKBP5* gene, which encodes the FK506 binding protein 51, a co-chaperone of the GR along heat shock proteins (hsp), including hsp90. The expression of FK506 is stimulated by glucocorticoids and has an inhibitory effect on GR signaling, preventing the nuclear translocation of GR. If short-lived, this negative feedback mechanism to reduce GC signaling may be important to restore HPA axis homeostasis. However, aging and certain stress-related phenotypes are associated with epigenetic up-regulation of *FKBP5* via decreases in DNA methylation at selected enhancer-related FKBP5 sites, promoting NF-κB–related peripheral inflammation and chemotaxis, and heightened cardiovascular risk (56). Importantly, translational research indicates that the type of response (regulated or dysregulated) is established early in critical illness (14, 31, 57), and the previously espoused hypotheses of the second-hit model (14, 22), or the two-phase model (compensatory anti-inflammatory response syndrome) are now both considered obsolete (29, 58, 59).

Based on this pathophysiological construct, we will focus on emerging evidence indicating the central role played by the activated glucocorticoid receptor-alpha (GRα), the master regulator of NF-κB and homeostatic corrections, in the development and resolution of critical illness. This role is conditioned by its interdependence with functioning mitochondria and by presence of adequate micronutrient reserves. Additionally, we present evidence on how CIRCI, mitochondrial dysfunction/damage, and hypovitaminosis negatively interact to accelerate an anti-homeostatic process of natural selection.

## Glucocorticoid Receptor-Alpha

The stress system is a complex, sophisticated, and carefully regulated adaptation mechanism that has been shaped by natural selection because it offers a selective advantage (4). All vertebrates express the proopiomelanocortin protein (POMC) that gives rise to adrenocorticotropic hormone (ACTH) which then stimulates the secretion of glucocorticoids. ACTH has long been closely associated with other signaling molecules, such as CRH, vasopressin, biogenic amines (epinephrine and norepinephrine), steroids such as cortisol and aldosterone, cytokines, such as IL-1β, and nitric oxide. All of these substances are crucial to adaptation to stressors. It is remarkable that the gene DNA sequences for these molecules have not only been conserved over hundreds of millions of years but also continue to serve closely related adaptive functions (4). This is apparently a result of strong selective forces against mutations that change their sequences and functionality of their products.

Cortisol, the end-product of HPA axis activation, is synthesized from cholesterol in the mitochondria and endoplasmic reticulum of the *zona fasciculata* of the adrenal cortex. Its synthesis depends entirely on scavenger receptor class B type-I (SR-BI)-mediated cholesteryl ester selective uptake from circulating high-density (HDLs) and low-density (LDLs) lipoproteins (60). In critically ill patients, low serum HDL levels correlate negatively with circulating TNF-α and IL-6 levels (61, 62), and positively with mortality (61, 63). The combined effects of reduced HDL and SR-BI during systemic inflammation may lead to significant reductions in glucocorticoid production (64). Low HDL (65) and low total cholesterol (66) correlate with inadequate response to ACTH stimulation. In septic shock, prolonged glucocorticoid administration is associated with significant increase in total cholesterol levels within 3 days of treatment (66).

**Figure 2:**
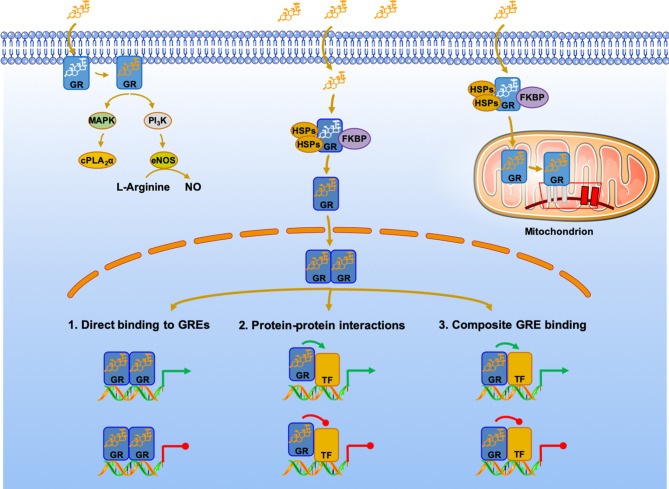
Genomic, non-genomic, and mitochondrial glucocorticoid signaling pathways. Glucocorticoid receptor mechanisms of action. The classic actions of GRα are shown in the middle of the panel. The dormant but ligand-friendly receptor, located in the cytoplasm, is bound to co-chaperon molecules, such as heat-shock proteins and the immunophilin FKBP. Upon binding to the ligand, the activated receptor translocates into the nucleus, where it interacts with GREs and/or other transcription factors, such as NF-κB and AP1, to regulate the activity of glucocorticoid-responsive genes, which represent ~20% of the human genome. In addition, cell membrane-associated GRα may be activated by the MAPK and PI3K kinases, as shown in the left of the panel. In addition, by as yet unknown mechanism, the GRα translocates into the mitochondria, where it interacts with the mitochondrial DNA GREs, regulating the activity of mitochondrial genes. See text for additional details cPLA2α, cytosolic phospholipase A2 alpha; eNOS, endothelial nitric oxide synthetase; FKBP, immunophilins; GR, glucocorticoid receptor; HSP, heat shock proteins; MAPK, mitogen-activated protein kinases; NO, nitric oxide; PI3 K, phosphatidylinositol 3-kinase; TF, transcription factor.

Glucocorticoids are the primary adaptive response mediators, whose signaling system interacts with other cell signaling systems, all essential for maintaining the homeostasis of many of the body's complex functions, including neural, cardiorespiratory, endocrine, metabolic, bioenergetic, and immune responses (67). Within tissues, glucocorticoids are regulated at the pre-receptor level by the isozymes of 11β- hydroxysteroid dehydrogenase (11β-HSD), which are located in the endoplasmic reticulum (ER). Glucocorticoids (GCs) bind to the ligand-binding domain of GRα to produce a biological response. Because of their lipophilic nature, GCs can readily diffuse across cellular membranes to bind to their intracellular receptor and produce a biological/pharmacological response [Figure 2; (15, 68, 69)]. The glucocorticoid receptor is a member of the nuclear receptor (NRs) (70) superfamily that emerged in the vertebrate lineage ~420–440 million years ago (similarly to NF-κB) from sequential duplications of two ancestral genes, those of the estrogen and the glucocorticoid receptors; the latter ultimately evolved into the glucocorticoid and the mineralocorticoid receptors (67, 71). Underlying its essential role in formation and regulation of multicellular life, the GR is required to establish the necessary cellular context for maintaining normal uterine biology and fertility through the regulation of uterine-specific actions (72) while GRs are vital for the structural and functional maturation of fetal organs (73, 74), affecting almost 4,000 genes (75). In late gestation of mammals, fetal glucocorticoid levels rise dramatically, an essential step for maturation in preparation for life after birth. Also, an association was found between greater maternal affection and warmth in early life and increased expression of glucocorticoid receptor genes in the offspring resulting in long-term health benefits (76).

GRs are present in the cytoplasm (68) and cell membrane (non-genomic effects) (77) in almost all cells of the body and in high concentrations (in neutrophils ~5,000; in macrophages ~10,000) (78). However, GR levels within the cell are not static, but are tightly controlled by numerous factors and at multiple levels. Notably, 16 variants of the human GR (hGR) have been identified to date with the potential of at least 256 combinations of homo- and hetero-dimers (68). Recent research indicates that the expression of different GR transcriptional and translational isoforms might be a significant factor determining how GCs influence the biological function and activity of specific cells and tissues (75). In contrast to GRα, the alternatively transcribed GRβ, which resides primarily in the cell nucleus, does not bind glucocorticoid, but can form homodimers with itself or heterodimers with a GRα subtype, functioning as an antagonist of GRα. GRβ homodimers can interact with glucocorticoid response elements (GRE) in the DNA, however their binding does not activate transcription (69). Generally, GRβ is expressed at very low levels compared to GRα; however, its expression is increased in inflammatory diseases, including critical illness, and this might be associated with decreased sensitivity to GCs and CIRCI (79).

Activation of GRα is not only an essential component of the general adaptation to stress, but also contributes to the maintenance of homeostasis in stress-free conditions (15). The biological response to the GC-GRα complex is affected by cell type, tissue type, and species-specific variations in the repertoire of partnering proteins, ligand concentrations, and other contextual variables (80, 81). In stimulated HeLa and neuronal PC12 cells, genome-wide studies identified 8,306 and 2,252 GR genomic binding sites upon treatment with GC, respectively (12, 81). Of interest, the availability of these binding sites for interacting with the GC-GR complex depends on the chromatin landscape, which is tissue- and cell type-specific, explaining to some extent, why the GR has a certain effect on one tissue and a totally different effect on another (67). Thus, even though the signaling system is the same, the landscape of the landing site is not. Thus, different cells recognize these signals differently, resulting in a highly context-dependent action by glucocorticoids (67).

After GC binding takes place in the cytoplasm, the activated GC-GRα complex can either (i) bind to several pro-inflammatory transcription factors, or (ii) act as a transcription factor, after translocation into the nucleus and mitochondria (69, 82). Glucocorticoids regulation of mitochondrial transcription via activation of mtGRE was the subject of a recent review (53). In pathway (i), the activated GC-GRα complex interacts directly with activated transcription factors NF-κB and AP-1, leading to the transcriptional repression of major downstream proinflammatory factors. In pathway (ii), GC-GRα binds to positive (transactivation) or negative (transrepression) specific DNA regions, the glucocorticoid-response elements (GREs) on the nuclear and mitochondrial DNA (83), to directly regulate transcription of target genes. Finally, GC activation of membrane-bound GR rapidly induces the activity of several kinases, such as the mitogen-activated protein kinase (MAPK) or the phosphatidylinositol 3-kinase (PI3K) pathways [Figure 2; (69)]. The non-genomic effects of GCs clearly differ from their well-known genomic effects, with the former responding within several minutes independently of protein synthesis. Genomic studies have shown that the GC-GRα complex regulates more than 3,000 genes in unstimulated peripheral blood mononuclear cells (PBMC) from healthy donors (84), in human pulmonary type II cells (85), and several organs, including the heart (86), liver (87, 88), and uterus (72) of unstimulated mice, underscoring its essential role as a master modulator in sustaining life and restoring health. The discordance between the number of regulated genes and the GR sites (12, 81) suggests that multiple sites are involved in the regulation of a single gene and/or that binding of a transcription factor is not sufficient to drive gene expression (12).

Control of mRNA turnover is critical in regulating the levels of inflammatory- and immune-mediated gene expression. Recent studies indicate that the GR can mediate GC actions beyond transcriptional gene control; it may actually directly participate, via association with mRNA, in GC-mediated control of cytoplasmic post-transcriptional mechanisms of gene expression (89). In an experimental model (SPRET/Ei mice), increased GR levels and activity were associated with strongly reduced expression levels of cytokines and chemokines in response to LPS-induced lethal inflammation (90).

## Glucocorticoid Receptor-Alpha and Homeostatic Corrections

**Figure 3:**
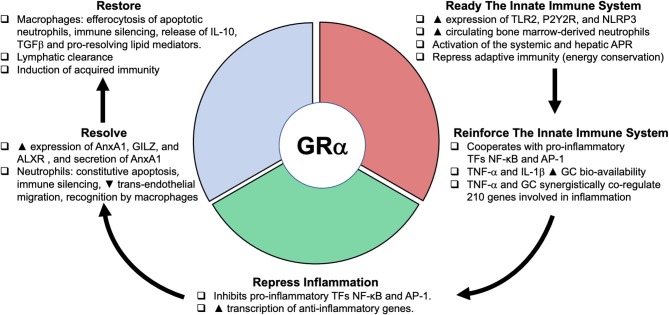
Glucocorticoid receptorα as cellular rheostat of homeostatic corrections. The glucocorticoid receptorα (GRα) acts as a cellular rheostat to ensure that a proper response is elicited by the neuroendocrine immune system throughout the three phases of homeostatic corrections: the pro-inflammatory (ready- reinforce), the anti-inflammatory (repress), and the resolution (resolve- restore) phase. Modified with permission from Busillo and Cidlowski (92). TLR2, toll-like receptor 2; purinergic receptor P2Y2R; NLRP3, NOD-like receptor pyrin containing 3; APR, acute phase response; TF, transcription factor; NF-κB, nuclear factor-κB; AP-1, activator protein 1; AnxA1, annexinA1; ALXR, AnxA1 receptor; GILZ, glucocorticoid-induced leucine zipper; TGFβ, transforming growth factor β.

In 1984, Munck et al. reviewed the actions of cortisol and proposed that “stress-induced increases in glucocorticoids levels protect, not against the source of stress itself, but rather against the body's normal reactions to stress, preventing those reactions from overshooting and threatening homeostasis (91).” This work and the results of the above genomic studies have led to a reevaluation of glucocorticoids' role in homeostatic corrections. Busillo and Cidlowski (92) recently reviewed the master regulatory role played the activated GC-GRα complex in the three major phases of homeostatic correction involved in disease development and resolution (Figure 3). While distinctions are made between the different states, variable degrees of overlap are likely. First, in the pro-inflammatory phase, GC-GRα prime the innate immune system to remove or neutralize pathogens by: (i) inducing the expression of Toll-like receptor 2, NOD-like receptor pyrin containing 3 (NLRP3) inflammasome, and purinergic receptor P2Y2R; (ii) repressing adaptive immunity (energy conservation); and (iii) cooperating with pro-inflammatory transcription factors NF-κB and activator protein 1 (AP-1) in promoting leukocyte redistribution. The GC induction of NLRP3 sensitizes the cells to extracellular ATP and significantly enhances the ATP-mediated release of proinflammatory molecules, including mature IL-1β, TNF-α, and IL-6 (93). In addition, inflammatory cytokines, particularly IL-6, nitric oxide, and GCs trigger and modulate the systemic and hepatic acute phase protein response (94). In stimulated HeLa cells, a genome-wide study identified 1,932 gene collectively regulated by the activation of NF-κB and GRα, with 43% of regulated genes responding only when both ligands are added, indicating that GRα and NF-κB crosstalk alters signaling pathways that are regulated by each factor separately (12).

During systemic inflammation, peripherally generated TNF-α, IL-1β, IL-6, and other inflammatory cytokines activate the HPA axis at multiple levels to produce GC (95–98). In addition, inflammatory cytokines increase the expression and enzymatic activity of 11β hydroxysteroid dehydrogenase type 1 (11βHSD1), which converts the inactive cortisone to the active cortisol in different cell types, as for example occurs after addition of TNF-α or IL-1β on endothelial (99) or lung epithelial cells (100). Thus, cytokines seem to amplify GC bioavailability (101). Microarray studies have shown that TNF-α and GC synergistically co-regulate 210 genes involved in inflammation (100). In this context, the synergy between GCs and pro-inflammatory cytokines is a useful mechanism for rapidly re-enforcing initial pro-inflammatory responses. Importantly, GC-GRα is necessary to prevent excessive phagocytic cell activation and improve intracellular bacterial killing (102).

In the second phase, when regulated systemic inflammation prevails, GC-GRα exerts classic anti-inflammatory action by (i) inhibiting NF–κB, AP-1 and other signaling pathways involved in inflammation, and (ii) increasing transcription of anti-inflammatory genes and the NF–κB inhibitory protein IκB (68, 103). GC-GRα anti-inflammatory action has been extensively investigated, and we direct the reader to excellent reviews on the (11, 68, 101, 104–106). In upcoming sections, we will review selected mechanisms involved in GC-GRα failure to downregulate systemic inflammation and achieve disease resolution.

The third phase involving disease resolution, i.e., restoration of tissue anatomy/structure and function, is an active and elegantly orchestrated process associated with multiple biochemical pathways, including switching production from pro-inflammatory to pro-resolving mediators (92). As downregulation of systemic and tissue inflammation continues, the activated GC-GRα engages in a host of pro-resolution mechanisms changing, among others, the phenotype of both granulocytes and macrophages. In these immune cells, via genomic mechanisms, GC-GRα increases the expression of AnnexinA1 (AnxA1), AnxA1 receptor (ALXR), and glucocorticoid-induced leucine zipper (GILZ), while via non-genomic mechanisms it increases the secretion of AnxA1 (107–109). The coordinated action of GILZ and AnxA1 is essential to regulating resolution (109). Granulocytes undergo constitutive apoptosis, disabling their potentially injurious secretion responses, i.e., NF–κB activation and transcription of inflammatory cytokines, and decreasing trans-endothelial migration leading to their rapid recognition and internalization by macrophages (efferocytosis) (109–112).

Apoptotic cells also serve as resolution cues for macrophages, which, after phagocytosis of apoptotic granulocytes, change their phenotype toward a more resolving/restorative one. The changes in phenotype from M1 (classically) to M2 (alternatively) leads to an orchestrated series of actions leading to successful resolution of inflammation. Interestingly, GCs promote phagocytosis (112, 113) and reduce the apoptotic granulocyte ingestion requirements for generation of M2 (114). GC-mediated change to M2 phenotype is associated with (i) immune silencing, where the release of inflammatory mediators and inducible nitric oxide synthase (iNOS) are suppressed (114); (ii) an increased release of the anti-inflammatory mediators IL-10 and TGFβ and several pro-resolving lipid mediators (114); (iii) protection from apoptosis; (iv) non-phlogistic degradation; (v) production of angiogenic growth factors; (vi) increased macrophage chemokinesis (by upregulation of genes involved in cell mobility) and lymphatic clearance; and (vii) induction of acquired immunity (110, 111, 113–117). GC-mediated Annexin 1-derived peptide (Ac_2−−26_) acting through the ALXR receptor has a pivotal role in the clearance of apoptotic cells (118). In models of self-resolving inflammation, various phenotypes of macrophages may coexist. In the later phase of resolution, M2 macrophages switch to the resolution-promoting macrophage (Mres) phenotype, which display reduced phagocytosis, while producing antifibrotic and antioxidant proteins that limit tissue damage and fibrosis (resolution of granulation tissue) (119). In human monocytes, GCs induce the expression of 133 genes with upregulation of anti-oxidation, migration, phagocytosis, and anti-inflammation with consequent downregulation of adhesion, apoptosis, and oxidation (113). In agreement with microarray data, spontaneous, as well as PMA-induced production of reactive oxygen species, was significantly reduced in GC-treated cells, and GCs promoted survival of an anti-inflammatory monocytic phenotype in inflammatory reactions (113).

## Glucocorticoid Receptor Alpha in Critical Illness

Since GRα ultimately controls GC-mediated activity, any condition that affects its concentration, binding affinity, transport to the nucleus, interactions with GREs (nuclear and mitochondrial), cofactor activity (see section Hypovitaminoses), or interaction of other relevant transcription factors (NF-κB, AP-1) and co-regulators, can eventually affect the response of cells to glucocorticoids (101, 120). The many different ways GRα function can be negatively influenced by the pro-inflammatory environment of critical illness was the subject of recent reviews (101, 121).

Critical illness-related corticosteroid insufficiency is a term used to define the central role played by the HPA-axis and the activated GRα complex in the pathogenesis of dysregulated systemic inflammation in critical illness (17). Three major pathophysiologic events account for the neuroendocrine decompensation observed in CIRCI: (i) multi-level dysregulation of the HPA-axis correlating with circulating inflammatory cytokine levels; (ii) altered cortisol metabolism [reviewed in (122)], and (iii) secondary generalized circulating and tissue specific reduction in GRα number/function with observed multifactorial tissue resistance to endogenous glucocorticoids (17). The role of mitochondrial oxidative stress in reducing GRα number/function is reviewed later (see section Oxidative Stress and CIRCI).

Experimental and clinical studies have demonstrated that critical illness is associated with a significant reduction in GRα density and transcription and an increase in GRβ-mediated dominant negative activity on GRα-induced transcription. In a human cell line, activation of NF-κB by TNF-α had a direct dose- and time-dependent effect on GR levels with a disproportionate increase in GRβ over GRα (123). Experimental sepsis is associated with decreased GRα transcription in circulating cells (124), heart (125), lymph node-spleen (124), liver (125–127), kidney (127), lung tissue (125–129), and skeletal muscle (125). Moreover, the endothelial GRα is required for protection against sepsis (see section Endothelium) (130). Importantly, the reduction of GRα expression is rapid and persists for at least 5 days (125) while it is associated with increased GRβ expression in the heart and lung but not liver (125), and increased NF-κB activation (127). Similarly, in experimental ARDS, lung tissue shows a significant reduction in GRα expression (128, 131, 132) and an increase in GRβ expression (131), leading to decreased GRα nuclear translocation (131). In transgenic mice, expression of GRα above wild-type levels leads to increased resistance to LPS-induced endotoxic shock (133).

**Figure 4:**
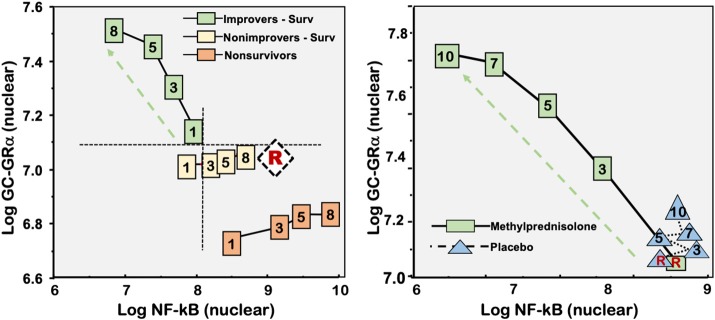
Correlation between mean levels of nuclear NF-κB and nuclear GC-GRα during the natural progression of ARDS, and in response to prolonged glucocorticoid treatment. Mean intracellular changes of nuclear GC-activated GRα and NF-κB observed by exposing PBLs of a healthy volunteer to plasma samples collected longitudinally (days 1, 3, 5, and 8) and after randomization to methylprednisolone treatment [randomization day (R) and post-randomization days 3, 5, 7, and 10]. The mean values of nuclear NF-κB are plotted against the mean nuclear GC-GRα levels. Improvers had a pre-defined improvement in lung injury score (139) and/or gas exchange component by day 7. The left panel shows ARDS patients with adaptive and maladaptive responses. In improvers, an inverse relation was observed between these two transcription factors, with the longitudinal direction of the interaction shifting to the left (decreased NF-κB) and upward (increased GC-GRα). In contrast, in non-improvers NF-κB increased over time while GC-GRα had no significant changes. We define the first interaction as GC-GRα-driven, and the second interaction as NF-κB-driven (33). The right panel shows non-improvers-survivors randomized after day 8 of ARDS to methylprednisolone (*n* = 11) vs. placebo (*n* = 6). After natural logarithmic transformation and adjustment for repeated measurements, partial correlations among responses to plasma from the methylprednisolone group were −0.92 (*p* < 0.0001) both for nuclear NF-κB and nuclear GRα. For responses to plasma from the placebo group, no significant relation was found between nuclear NF-κB and nuclear GRα (*r* = 0.11; *p* = 0.70) (103). Reproduced with permission from Meduri et al. (11).

Clinical studies, including autopsies, in patients with severe sepsis and septic shock have reported a significant reduction in GRα expression in circulating cells (79, 134–137); heart (125), liver and skeletal muscle (125, 138), and a significant increase in GRβ expression in the heart and liver (125). GRα mRNA in neutrophils correlates negatively with plasma IL-6 levels and shows gradual recovery overtime in survivors (135). In another study, neutrophil GRα mRNA levels decreased 4-fold by day 4 in the ICU and remained low for 2 weeks (79). In *ex vivo* experiments with PBMCs exposed to longitudinal plasma samples from patients with ARDS results suggested that insufficient GC-GRα-mediated activity at disease onset and over time was a central mechanism for the upregulation of NF-κB activity (Figure 4). Over time, patients with regulated systemic inflammation have a progressive increase in all measured GC-GRα-mediated activities, including GRα number, binding to NF-κB and to nuclear GRE, as well as increased transcription of IκBα and IL-10, and a corresponding reduction in NF-κB nuclear binding, and transcription of TNF-α and IL-1β. In contrast, patients with dysregulated systemic inflammation had only a modest longitudinal increase in GC-GRα-mediated activities and a progressive increase in NF-κB nuclear binding that was most striking in non-survivors (33). By day 3 of ARDS, no overlap was observed between groups for NF-κB and GC-GRα nuclear binding. In lung tissue obtained by open lung biopsy, histological severity correlated with increased nuclear uptake of NF-κB and a lower ratio of GRα: NF-kB uptake (33). A decrease in GRα expression in critical illness is maladaptive granted that proinflammatory pathways are not properly restrained (124).

Randomized studies (103, 129, 132) demonstrated in both circulating and tissue cells, that quantitatively adequate and prolonged glucocorticoid supplementation increased GRα number and function, reversing critical illness-associated cellular glucocorticoid resistance. In experimental ARDS, low-dose glucocorticoid treatment restored GRα number and function leading to resolution of pulmonary inflammation (129, 132). Similarly, in an *ex vivo* ARDS study, prolonged methylprednisolone treatment was associated with upregulation in all measurements of GRα activity leading to reduction in NF-κB DNA-binding and transcription of inflammatory cytokines [Figure 4; (103)]. Glucocorticoid treatment changes the longitudinal direction of systemic inflammation from dysregulated (NF-κB-driven, maladaptive response) to regulated (GRα-driven, adaptive response) with significant improvement in indices of alveolar-capillary membrane permeability and markers of inflammation, hemostasis, and tissue repair (11).

## Endothelium

The vascular endothelium constitutes the innermost lining of the body's circulatory system and the largest tissue in the body (close to 100,000 miles long) containing ~2.5 × 10^12^ endothelial cells that are typically flat and susceptible to injury, with a thin basement membrane enriched in type IV collagen and laminin (140). The endothelial lining is in continuous contact with circulating cells and soluble proteins, and the capillaries, represent the primary barrier between elements in the blood and the parenchymal cells. The space between two contiguous endothelial cells, known as the endothelial cleft (ETC), acts as an important site of regulation of endothelial (paracellular) permeability (141). Importantly, the vascular endothelium (micro- and macro-circulation) is clothed with a protective barrier, the glycocalyx, which is critical to maintain endothelial homeostasis. The endothelial glycocalyx is a negatively charged, organized mesh of membranous glycoproteins and plasma proteins that include superoxide dismutase, antithrombin III, and cell adhesion molecules, all involved in maintaining the oncotic gradient across the endothelial barrier (141). The intact glycocalyx protects endothelial cells from oxidative stress and prevents the interaction between circulating leukocytes and endothelial adhesion molecules (141). Conformational changes in glycocalyx structure lead to short bursts in the release of endothelial nitric oxide (eNO) (141), inhibiting vascular smooth muscle contraction, platelet aggregation, and leukocyte adhesion, all three processes essential for patency of microcirculation. The blood-brain barrier (BBB), composed of highly specialized endothelial cells with tight junctions that seal paracellular spaces to restrict permeability, serves as a highly restrictive interface between the systemic circulatory system and the brain (142).

Damage to the glycocalyx precedes vascular pathology. Endothelial activation with ubiquitous shedding of the glycocalyx is a major component of critical illnesses and a key pathogenic mechanism in multiple organ dysfunction. The pathways by which sepsis induces injury to the endothelium were recently reviewed (143). The “vasculo-centric view” of critical illness derives from the observation that, despite the remarkable heterogeneity of diseases, the pathobiology of multiple organ dysfunction shares near-stereotypical features that are mostly related to widespread endothelial dysfunction (144). Endothelial dysfunction manifests with a diffuse increase in paracellular permeability, expression of luminal cell adhesion molecules, recruitment of activate leukocytes, altered vasomotor tone, and microvascular thrombosis with decreased capillary density (145). Increasing evidence points to endothelial dysfunction with impairment of the BBB as a critical component of the pathobiology of delirium during critical illness (146).

Oxidative stress (see section Mitochondrial Cacostatic Load, Oxidative Stress, and Mitochondrial Damage) impairs endothelial function by interfering with eNO synthesis (147) and by participating in the degradation of the glycocalyx (141). After shedding of the glycocalyx, adhesion molecules are released in the blood and can be found in the circulation (35). Microvascular alterations, such as decreased functional capillary density and increased perfusion heterogeneity, are frequently observed in patients with sepsis and contribute to the defect in oxygen extraction by the peripheral organs and tissues of the organism (148).

Endothelial activation may also affect the HPA-axis. The adrenal gland is a highly vascularized organ, with every steroidogenic cell in close vicinity with at least one sinusoid, and a clear positive relation between adrenal blood flow and steroidogenesis has been demonstrated (149). In critical illness, disruption of endothelial homeostasis within the adrenal gland can contribute to the HPA-axis dysfunction (150). Hypovitaminosis may also contribute to endothelial dysfunction (see section Hypovitaminoses).

In studies of circulating inflammatory cytokines, there is substantial evidence that in critically ill patients, an increase in circulating markers of endothelial integrity (angiopoietin-1; Angpt-1) (145), dysfunction [angiopoietin-2 (Angpt-2), von Willebrand factor (VVF) (150), soluble intercellular adhesion molecule-1 (sICAM-1), (35) vascular endothelial growth factor (VEGF)] (145, 151), and cell damage associated with circulating endothelial cells (152, 153) correlate with disease severity and mortality. Fittingly, a large study acquiring sublingual measurements of microcirculation in early sepsis, found that mortality strongly correlated with the severity of alterations in the proportion of perfused small vessels, i.e., the functional capillary density (154).

The endothelial GRα is a critical regulator of vascular homeostatic corrections and essential for decreasing the rolling on and adhesion of activated neutrophils to the endothelium (155). In experimental sepsis, elimination of the endothelial GRα resulted in prolonged activation of endothelial NF-κB, with increased expression of iNOS and inflammatory cytokines, both accounting for hemodynamic collapse and mortality (130). Importantly, the presence of the endothelial GR itself was necessary for GC-mediated suppression of NF-κB and for achieving survival (130). GRα also regulates the tightness of the BBB, inducing expression of the tight junction proteins occludin and claudin-5, and the adherens junction protein vascular endothelium cadherin (VE-Cadherin) (156).

GC-GRα is also strongly involved in vascular development (81). Experimental studies have shown GR-dependent upregulation of multiple mediators involved in endothelial cell homeostasis, such as sphingosine kinase 1 (SphK1) (157), angiopoietin-1 (Angpt-1) (158), serum glucocorticoid kinase-1 (SGK-1) (159, 160), GILZ (161), and eNOS (162–164). In experimental ARDS, upregulation of SphK1, an important regulator of endothelial barrier integrity, was shown to improve alveolo-capillary membrane (ACM) permeability (157). In human brain microvascular endothelial cells (HBMECs), GC treatment was associated with transcriptional activation of Angpt-1 and suppression of VEGF (158). In umbilical vein endothelial cells (HUVECs), upregulation of SGK-1 reduced oxidative stress and improved cell survival and senescence (159); meanwhile, GR-induced GILZ expression (see section Glucocorticoid Receptor-Alpha and Homeostatic Corrections) correlated negatively with vascular inflammation (161). In neuro-vascular tissue, physiological doses of hydrocortisone rapidly activated eNOS via non-genomic mechanisms (163, 164).

GRα is also a critical regulator of myocardial function. In experimental work, the GR—via Kruppel-like Factor 13—was found to play a direct role in the regulation of cardiomyocyte function and protection from hypoxia and DNA damage (86). GR inhibits cells death triggered by ischemia reperfusion, mechanical stress, or TNFα [reviewed in (86)].

The endothelial response to GCs in inflammatory diseases was extensively reviewed covering topics such as inhibition of pro-inflammatory transcription factors, restoration of endothelial barrier integrity, and induction of protective molecules (140). In experimental sepsis, low-dose glucocorticoid (hydrocortisone or dexamethasone) preserved the endothelial glycocalyx, sustained the vascular barrier and reduced interstitial edema (165, 166), and had beneficial effects on mesenteric blood flow and helped with resolution of organ injury (167). GCs play an important role in the control of vascular smooth muscle tone by their permissive effects in potentiating vasoconstrictive responses to catecholamines and other hormones, such as arginine-vasopressin, through glucocorticoid receptors (168). Finally, they inhibit the expression of inducible nitric oxide synthase and other vasodilatory agents in vascular endothelial cells (169). Additional experimental studies have shown that GR stimulates transcription of the *zonula occludens* (ZO)-1 tight junction protein, leading to reduced BBB paracellular permeability (142), while it activates eNOS increasing cerebral blood flow (163).

In patients with septic shock (170, 171) or ARDS (172, 173), prolonged glucocorticoid (hydrocortisone or methylprednisolone) treatment resulted in the following: (i) increased plasma activated protein C levels (173); (ii) reduction in markers of endothelial injury such as sICAM-1 (35); (iii) rapid and consistent improvement in capillary perfusion, independently of the cortisol response to ACTH (170); and (iv) improvement in alveolar-capillary (172) and renal (171) endothelial permeability. In addition, septic shock is associated with vascular dysfunction through NF-κB-mediated downregulation of the endothelial mineralocorticoid receptor (MR) and α1-adrenoceptor, which can be restored with mineralocorticoid (fludrocortisone) treatment (174).

## Cellular Energetics— Mitochondrial Function

A transforming event in the history of life was the evolution of photosynthetic bacteria, with biochemical pathways that allowed them to capture energy from sunlight and store it in simple sugars, a process known as photosynthesis that generates oxygen as a waste product. As a result, over the course of about one billion years, the earth's atmospheric oxygen increased from almost zero to nearly modern levels (175). The development of an ozone layer in the upper atmosphere to absorb damaging UV radiation from the sun, a derived outcome of increased atmospheric oxygen, permitted organisms to live on land for the first time (175). Some groups of organisms adapted to increased oxygen levels; the most notable adaptation was the evolution of the biochemical pathways of cellular respiration, which use oxygen to extract the energy stored in organic molecules much more efficiently.

About 2 billion years ago, complex life surfaced with two major endosymbiotic (eukaryotic cells ingesting a prokaryote bacterium that resulted in a symbiotic relationship between the engulfing and engulfed cells) events igniting the evolutionary progression to animals and plants (176, 177). First, the ancestral eukaryotic cell engulfed an aerobic prokaryote bacterium (i.e., capable of using oxygen to produce energy) that eventually evolved into mitochondria (specialized for cellular respiration) populating the cell cytoplasm (modern heterotrophic eukaryote) to afford a selective advantage for survival (178). In the second endosymbiotic event, the early eukaryotic cell engulfed a photosynthetic prokaryote bacterium that evolved into the chloroplast (modern photosynthetic eukaryote).

Central to the integrated actions of immune and neuroendocrine responses (3, 17, 179) is cellular energetics, involving the mobilization of energy resources from GC-GRα-mediated breakdown of glucose (via glycolysis), fatty acids and amino acids for mitochondrial energy production (180). In fact, GCs were originally named by Hans Selye based on their ability to increase blood glucose concentration. Activation of the HPA axis mobilizes these energetic substrates into the circulation within minutes, underscoring the widespread role of GC-GRα in the regulation of systemic metabolism (181).

Mammalian cells, apart from erythrocytes, contain within their cytoplasm hundreds to thousands of mitochondria, the number determined by the energy demand of each cell type. Mitochondria are autonomous and highly dynamic double-membrane organelles that function as the powerhouses of the cell and utilize ~98% of total body oxygen consumption. Oxidative phosphorylation (OXPHOS) is the metabolic pathway in the inner membrane of the mitochondria which use enzymes to oxidize ingested calories to produce adenosine triphosphate (ATP) required for normal cell functioning. Ultimately, this conversion provides energy for most cellular processes within the body intracellular reactions (gene transcription and translation, epigenetic modifications), hormonal changes in the endocrine system, structural changes in tissue, and behavioral and cognitive responses] (181), and in theory, determines the limits of an organism adaptive capacity (21).

By virtue of their origins as aerobic bacteria, mitochondria have their DNA, RNA, and protein synthesis systems. The mitochondrial DNA (mtDNA) in our proto-eukaryotic ancestors was significantly larger in genetic complement, but transfer of mtDNA encoded genes to the nucleus has occurred over the 1.5–2 billion years since the origin of the eukaryotic cell, and today the mammalian mtDNA (inherited from the mother) encodes 13 polypeptides, two rRNAs (12S and 16S) and 22 tRNAs that are essential for OXPHOS and proper cell function (178). Since the mtDNA encodes for only a handful of proteins, mitochondria depend on the cell nucleus and other cellular compartments for most of their proteins and lipids (182). In addition to being the major source of intracellular ATP, mitochondria are deeply involved in signaling pathways, elicited by perturbations in homeostasis, which promote cell adaptation (183, 184).

Mitochondria constantly generate reactive oxygen species (ROS) as a by-product of substrate oxidation and oxidative phosphorylation, a physiological process that is normally kept in check by a diversified set of antioxidant defenses (184). The introduction of oxygen to earth's early biosphere stimulated remarkable evolutionary adaptations, and in this context, ROS should be viewed as an essential consequence and driver of evolution and survival over time (185). Reactive oxygen species are required in numerous physiological cell functions, such as cellular signaling systems linked to the transcriptional machinery, maintenance of vascular tone, oxygen sensing, and host defense against pathogens (186). One of the mitochondrial oxidases, the NADPH oxidase of polymorphonuclear leukocytes (primarily neutrophils), is pivotal to the body's defense against pathogenic microorganisms (187).

Mitochondria are involved in a multitude of cellular processes, well-beyond their long-established role as the cell's powerhouse. These include processes such as intracellular calcium homeostasis (buffering of cytosolic calcium), regulation of mitochondrial metabolism, cell migration, production of biomolecules such as amino acids, lipids, hemes, purines, and steroid hormones (see section Mitochondria and HPA-Axis Cross-Talk), and activation of cell death pathways (188). Mitochondria trigger cell death pathways by two mechanisms, first via necrosis when ATP levels fall below a certain threshold, and second via apoptosis through the release of mitochondrial cytochrome c into the cytoplasm (189). Mitochondrial integrity is, therefore, essential for the function and survival of cells, and several recent publications have highlighted the critical role played by these organelles in sustaining homeostatic corrections (82, 180–182, 190–192).

## Mitochondrial Cacostatic Load, Oxidative Stress, and Mitochondrial Damage

In critical illness, tissue oxygen consumption and total energy expenditure are increased, with intracellular metabolism boosted by up to 200% compared to the healthy state (193). Cells that represent the innate immune system, like neutrophils, and macrophages, are mainly responsible for the oxidative burst that takes place early in critical illness (194) along with the generation of ROS and nitrogen oxygen species (RNS) that are important for host defenses. In neutrophils of critically ill patients, oxidative activity correlates positively with the degree of intranuclear NF-κB expression (195). According to the “mitohormesis theory,” when present in moderate amounts, ROS and RNS function as intracellular signaling molecules that may improve systemic defense mechanisms by inducing an adaptive response (196). However, when intracellular ROS and RNS concentrations overwhelm antioxidant defenses, cell homeostasis becomes compromised (196). For example, peroxidation of the mitochondrial lipid cardiolipin in the inner mitochondrial membrane leads to dissociation of cytochrome c, reduced production of ATP, and increased generation of ROS (197).

**Figure 5:**
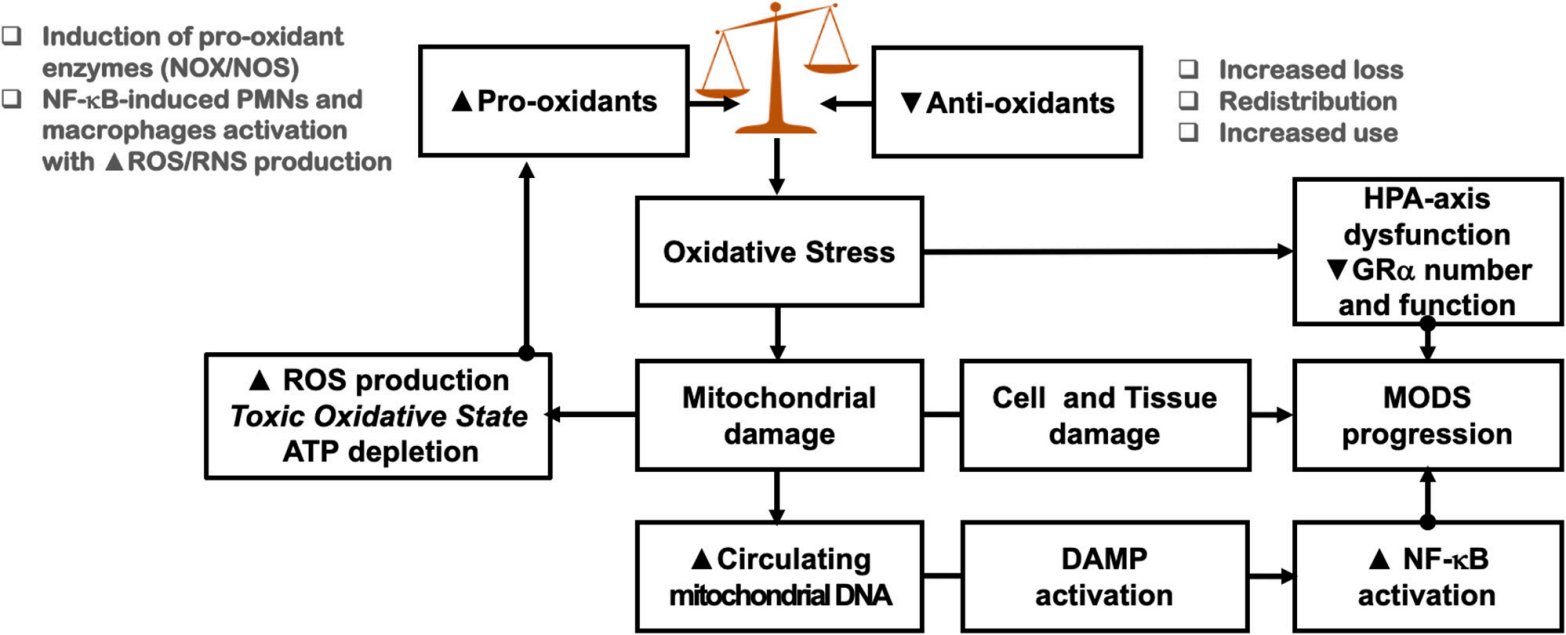
Impact of oxidative stress on homeostatic corrections. See sections Mitochondrial Cacostatic Load, Oxidative Stress, and Mitochondrial Damage, Mitochondria and HPA-Axis Cross-Talk, and Oxidative Stress and CIRCI for explanation. PMN, polymorphonuclear cells; MODS, multiple organ dysfunction syndrome.

Increased energy and metabolic demands associated with NF-κB-driven dysregulated systemic inflammation, leads to overproduction of ROS and RNS, resulting in significant damage of lipids, proteins, and nucleic acids, both within the mitochondria and in other compartments of the cell [Figure 5; (197)]. Oxidative stress is a predictor of mortality in septic shock patients (198). Glutathione is one of the most important redox buffers of the cells, as it can be found in all cell compartments and acts as a cofactor of several enzymes, helps in DNA repair, scavenges ROS (e.g., hydroxyl radicals, hydrogen peroxide, and lipid peroxides), and generates other antioxidants, such as ascorbic acid (see section Hypovitaminoses) and tocopherols (194). Vitamins D and C upregulate glutathione synthesis and prevent depletion (see section Hypovitaminoses) (199, 200).

The multi-level organization of mitochondrial molecular composition, structures, functions, and signaling roles within the cell was recently reviewed (201). In laboratory models of sepsis, mitochondrial respiration is often increased in the early phase of illness, but consistently falls with protracted inflammation [reviewed in (202)]. Many clinical studies implicate mitochondrial dysfunction and bioenergetic failure as an important pathophysiological mechanism underlying dysregulated systemic inflammation-associated multiorgan dysfunction. In a study of skeletal muscle biopsies obtained in septic patients, an association was first reported between nitric oxide overproduction, antioxidant depletion, mitochondrial dysfunction, and decreased ATP concentrations, with progression of multiorgan failure and mortality (203). Human septic cardiomyopathy is accompanied by widespread down-regulation of cardiac mitochondrial genes and decrease in the expression of genes that govern cardiac myocyte contractility, analogous to the transcriptional reprogramming that occurs in myocardial hibernation (204). Early sepsis is associated with a reduction in PBMC mitochondrial copy number, and a rise in markers of mitochondrial damage, in a linear relation to proinflammatory cytokine expression (205). The PBMCs of patients with severe vs. less severe pneumonia have increased ROS density, increased DNA damage, and decreased superoxide dismutase (SOD) concentrations (206). Loss of mitochondrial function may lead to compensatory secondary metabolism, glycolysis, to produce ATP as well as lactate (207). A epithelial cell line study demonstrated that this glycolytic switch is promoted by the activation of the redox sensitive phosphoinositide 3-kinase (PI3K) pathway and subsequent inactivation of glycogen synthase kinase-3β (GSK-3β) resulting in increased production of inflammatory cytokines and reduced sensitivity to glucocorticoids (207).

Decades of laboratory and clinical studies have revealed that dysregulated systemic inflammation, including sepsis, is associated with significant macromolecular damage to mitochondria, particularly in the cells of highly metabolically active tissues, such as the liver, heart, kidneys, brain, and skeletal muscles (191, 208). In contrast to nuclear DNA, which is non-immunogenic, mitochondrial DNA resembles bacterial DNA (see section Cellular Energetics—Mitochondrial Function) and acts as a damage-associated molecular pathogen (DAMP) to activate immune responses through Toll-like receptor 9-mediated activation of NF-κB (209) and the NLRP3 inflammasome (210). Also, in comparison to nuclear DNA, mtDNA is 50-fold more sensitive to oxidative stress (191), as its close proximity to the electron transport chain and the absence of chromatin proteins makes it an easy target for oxidative damage (197).

As a consequence of increased ROS generation, mtDNA can undergo several qualitative and/or quantitative alterations. Recent studies have found that critically ill patients have decreased cellular mtDNA levels and increased circulating cell-free mtDNA levels (205, 210, 211). In septic patients, mtDNA depletion in circulating cells (mainly neutrophils) correlates with severity of illness (APACHE II scores) (212), while high TNF-α expression in neutrophil lysates correlates with increased plasma mtDNA levels (205). The exact mechanism of mtDNA delivery into the cytoplasm and then into the systemic circulation is currently unknown (189). In ICU patients with sepsis and ARDS, elevated plasma mtDNA levels (>3,200 copies/μl) are associated with dysregulation of phospholipid metabolism (211), and increased mortality (210).

The cycle of mtDNA damage with loss of function of electron transport enzymes (ATP depletion) and increased ROS generation, a state in which the antioxidant systems are overwhelmed may eventually lead to cell death, a phenomenon known as the “*toxic oxidative stress*” (213). In critical illness, impaired cell energy metabolism and mitochondrial damage augment systemic inflammation directly via NF-κB activation and indirectly by multi-level impairment of the HPA axis and GR homeostatic functions (see section Oxidative Stress and CIRCI). Effective homeostatic corrections in the adaptive response during the resolution of critical illness are associated with increased mitochondrial biogenesis, restoration of oxidative metabolism, and mitochondrial content (205). In many studies, restoration of mitochondrial homeostatic functions was observed in association with improved organ recovery and survival [reviewed in (205)]. The association between mitochondrial dysfunction, circulating cell-free mtDNA, muscle wasting, sterile inflammation, and inflamm-aging was recently reviewed (189).

Micronutrient deficiencies may also impair mitochondrial function. In septic patients, the marked early reduction in selenium levels may affect selenium-dependent anti-oxidants glutathione peroxidase and thioredoxin (214). The role of hypovitaminoses on mitochondrial function is reviewed in section Hypovitaminoses.

## Mitochondria And HPA-Axis Cross-Talk

Abundant mitochondria are one of the most prominent ultrastructural features of the adrenocortical cells (208) in which intracellular steroidogenic cholesterol is ultimately converted to cortisol in a tightly controlled manner (82). The central role of mitochondria in essential physiological processes has rendered these organelles a receiver and integrator hub of multiple regulatory signals (215). Mitochondria participate in the stress response, in part, by sensing levels of glucocorticoids (183). It is now accepted that mitochondria are under GC control because GRs are present in mitochondria, and GREs reside in the mitochondrial genome (82, 192). A number of studies in several tissues have observed a cytoplasmic-to-mitochondrion GR translocation or vice versa in response to GC, indicating that mitochondrial GR is dynamically regulated upon exposure to GCs (69). Lee and collaborators have classified five pathways in the functional modulation of the mitochondria by GC-GR (82). In addition to direct mitochondrial GR-mtGRE interactions, mitochondrial gene expression is regulated indirectly by nuclear GR-nuGRE interactions that result in increased transcription of the following: (i) genes encoding OXPHOS and other mitochondrial regulatory functions, (ii) transcription factors for control of nuDNA-encoded mitochondrial proteins, and (iii) several antioxidant mechanisms including uncoupling protein-2 (UCP2) (69, 82, 113, 192, 216, 217). Of interest, mitochondrial thioredoxin, an antioxidant and anti-apoptotic factor essential for cell viability and vascular homeostasis (218), interacts directly with both the ligand and the DNA-binding domains of GR, keeping the receptor in a reduced, transcriptionally active form (219, 220).

Studies have shown that fine-tuning of the response to cellular demands is coordinately regulated by the nucleus and mitochondria, making mitochondrial-nuclear interaction vital to optimal mitochondrial function (82), with GC-GR-mediated increased mtDNA gene expression augmenting the total number of mitochondria per cell, and, thus, total cellular energy production capacity (216). Altogether, there is substantial evidence that cross-talk between neuroendocrine control of the stress response and cellular antioxidant systems is essential for mammalian homeostatic regulation (220). Consistent with the cacostatic load model (21), administration of physiological doses of GCs acutely increases mitochondrial membrane potential, calcium buffering capacity, anti-oxidant capacity, and resistance to apoptotic signaling (8), whereas chronic exposure to high doses of GCs suppresses anti-oxidant capacity, decreases mitochondrial membrane potential, and sensitizes cells to apoptosis (21, 187, 190).

## Oxidative Stress And CIRCI

Oxidative stress is accompanied by multi-level impairment of the HPA axis and GR homeostatic functions (Figure 5). In non-survivors of septic shock, marked overexpression of iNOS in hypothalamic parvocellular neurons (PVN) was associated with decreased expression of pituitary ACTH, suggesting that the pro-apoptotic action of iNOS in the PVN may partially account for reduced activity of the HPA axis in sustained septic shock (221, 222). In experimental sepsis, adrenal cellular extracts demonstrate a pronounced increase in mRNA for iNOS and inflammatory cytokines that correlate positively with the degree of neutrophil infiltration, adrenal cell apoptosis, and mortality (213).

Changes within the adrenal gland microenvironment may also affect the HPA axis response in critical illness (149), with mitochondrial damage leading to a decreased responsiveness to ACTH (208). Importantly, iNOS expression in adrenal cells diverges at 48 h, with a significant increase observed in non-survivors vs. a reduction in survivors (223). In experimental endotoxemia, NF-κB-mediated iNOS release is associated with mitochondrial oxidative stress in adrenocortical cells with inhibition of steroidogenesis and response to ACTH (208).

Oxidative stress has a direct deleterious impact on GRs number and function. Experimental studies involving tissue cultures (220, 224–226) and murine models (227, 228) have demonstrated that oxidative stress is associated with decreased: (i) GR number (228), (ii) GC binding to GR (220, 224–227), (iii) GC-GR nuclear translocation (226, 229), (iv) binding to DNA (224), and (v) inducible gene transcription (220, 225). Nitrosylation, the covalent incorporation of a nitric oxide “nitrosyl” moiety into the critical cysteine(s) residue(s) of the GR is associated with loss of the steroid binding capacity (230).

In human monocytes, genes involved in oxidative functions were significantly overrepresented among GC down-regulated genes, while genes with antioxidant functions were upregulated (113). A few studies have evaluated the impact of GC treatment on oxidative stress. In human monocytes, spontaneous, as well as phorbol myristyl acetate (PMA)-induced production of reactive oxygen species, is significantly reduced in GC-treated cells in comparison to controls (113). In murine macrophages, glucocorticoid treatment is associated with rapid (non-genomic) inhibition of superoxide anion production (231). In murine sepsis, GC treatment attenuated renal dysfunction by reducing mitochondrial injury with preservation of cytochrome c oxidase and suppression of pro-apoptotic protein levels (232). In clinical (233, 234) and experimental (162, 235) randomized trials, participants with severe sepsis receiving GC treatment had, in comparison to controls, a significant reduction in (i) circulating nitric oxide levels (162, 233, 235), and (ii) spontaneous release of hydrogen peroxide (H_2_O_2_) by neutrophils (234).

## Hypovitaminoses

Metabolic homeostasis is substantially disrupted in critical illness, and the degree of a vitamin deficiency can negatively impact health outcomes. Three vitamins, namely thiamine (vitamin B1), ascorbic acid (vitamin C), and vitamin D, are important for the proper function of the GR system and mitochondria, and their reserves are rapidly exhausted in critical illness (236). Vitamins B1, C, and D impact mitochondrial function, while vitamins C and D also impact GR function. A comprehensive list of suggested mechanisms for the efficacy of thiamine, ascorbic acid, and glucocorticoids in sepsis was recently reviewed (237).

### Thiamine

Thiamine is a water-soluble vitamin, which is passively absorbed in the small intestine. After ingestion, free thiamine is converted to the active form thiamine pyrophosphate (TPP), commonly known as vitamin B1, by thiamine pyrophosphokinase. The majority of TPP in the body is found in erythrocytes and accounts for ~80% of the body's total storage (238). Thiamine pyrophosphate is a key co-factor for pyruvate dehydrogenase, alpha-ketoglutarate dehydrogenase, transketolase, and branched-chain keto-acid dehydrogenase (238). Pyruvate dehydrogenase is the gatekeeper for entry into the Krebs Cycle, without which pyruvate would be converted to lactate as opposed to acetyl-coenzyme A. Alpha-ketoglutarate dehydrogenase is required for completion of the Krebs Cycle once it has begun. Transketolase is a key enzyme for the pentose phosphate pathway and for the production of NADPH with glutathione cycling, an important anti-oxidant pathway (239). There are also other proposed non-cofactor roles of thiamine within the immune system, gene regulation, oxidative stress response, cholinergic activity, chloride channel function, and neurotransmission (238).

The human adult can store around 30 mg of thiamine in muscle tissue, liver and kidneys, however, these stores can become depleted in as little as 18 days after the cessation of thiamine intake (238). A thiamine deficiency syndrome, beriberi, bears a number of similarities to sepsis, including peripheral vasodilation, cardiac dysfunction, and elevated lactate levels (237). In critical illness, the prevalence of thiamine deficiency is 10–20% upon admission (198, 240) and can increase up to 71% during ICU stay, suggesting rapid depletion of this vitamin (198). Based on limited data, no association was detected between thiamine levels, markers of oxidative stress (198) and mortality (198, 241). In one study, a significant negative correlation was reported between thiamine and lactic acid levels in patients with sepsis without liver dysfunction (240). In a pilot randomized controlled trial (RCT) of patients with septic shock (*n* = 88), the administration of thiamine (200 mg twice a day for 7 days) reduced lactate levels and improved mortality over time in a pre-defined subgroup of patients with thiamine deficiency (35% of cohort) (239). In a retrospective, single-center, matched cohort study, administration of thiamine within 24 h of septic shock (*n* = 123) was associated with improved likelihood of lactate clearance and a reduction in 28-day mortality (242). Despite some promising results, there is insufficient evidence to support or reject thiamine supplementation as a monotherapy in critically ill patients (238).

### Vitamin D

Vitamin D is an ancient molecule that functions as both a nutrient and a hormone with metabolic and immunomodulatory properties; it regulates over 1,000 genes of the human genome (243, 244). The vitamin D receptor (VDR) is a member of the nuclear receptor gene family and is expressed in virtually all nucleated cells. Decreased serum levels of vitamin D have been associated with several autoimmune inflammatory diseases. Genome-and transcriptome-wide studies indicate that vitamin D signaling modulates many inflammatory responses on several levels (245), including interference with NF-κB, via upregulation of IκB expression (246). In addition, the ability of vitamin D to inhibit metabolic stress and energy expenditure in a cell microenvironment suggests that this pleiotropic hormone has a broad task as a pro-survival agent (244).

A growing body of scientific and medical literature supports the important anti-inflammatory functions of vitamin D in health and disease, including the enhancement of GC-mediated anti-inflammatory actions (247). The anti-inflammatory effect of vitamin D was consistently observed in studies of cell lines and human PBMCs, and was the subject of a comprehensive review (248). PBMCs activated with TLR ligands after incubation with 1,25(OH)D3 showed decreased release of TNF-α and IL-1β and increased anti-bacterial activity (249). In PBMCs, physiologic levels of vitamin D reduce inflammatory activities, by upregulating GC-mediated mitogen activated protein kinase (MAPK; see section Glucocorticoid Receptor-Alpha) phosphatase-1 (MKP-1) (250) to down-regulate p38 MAPK-mediated inflammatory gene expression (including TNF-α, IL-1β, IL-6, and IL-8) (251). In LPS-activated PBMCs (247) and PBMCs from patients with asthma (252), vitamin D enhanced dexamethasone-induced expression of MKP-1 (247), and this synergism was dependent on vitamin D-induced GM-CSF release (247). One study suggested that the interaction between vitamin D, glucocorticoids and their cognate receptors is related to the duration of exposure to vitamin D (253).

Beside this indirect modulation of signaling cascades, vitamin D and its receptor complex VDR/RXR can interact directly with the GC receptor and other transcription factors (245). Of interest, Vitamin D has a high affinity binding for the GR (254), and was recently shown to increase, in a dose- dependent manner, GR concentration in T cells (255). Based on its pleiotropic functions, vitamin D is considered a “master tuner” in shifting homeostatic balance from a pro-inflammatory to a pro-resolving status (244). Several studies demonstrated a dose-dependent response of vitamin D with respect to reducing inflammation, with 1 nM and 10 nM concentrations causing the greatest effects (248). One study showed that serum 25(OH)D levels as high as 120 nmol/l may be necessary for optimal immune function (256). A small study in healthy patients with hypovitaminosis D reported that significant anti-inflammatory benefits of vitamin D supplementation were only seen by achieving serum 25(OH)D levels greater than 100 nmol/l (256).

The mitochondria also appear to be a direct target of the vitamin D endocrine system, and the two most important enzymes responsible for activation or inactivation of 25(OH)D, namely CYP27B1 (1α-hydroxylase) and CYP24A1 (24-hydroxylase), are located in the mitochondria (257). In U937 monocytes, 1,25 (OH)2 vitamin D upregulates glutamate cysteine ligase (GCLC) and glutathione reductase (GR), resulting in an increase of cellular glutathione formation, and decreased ROS and IL-8 secretion (258). Two recent studies have evaluated the impact of vitamin D on skeletal muscle mitochondrial function. Primary human skeletal muscle cells treated with 1,25(OH)D3 vs. vehicle demonstrated marked effects on mitochondrial number, morphology, physiology, and expression of key mitochondrial proteins, resulting in increased ATP production (259). In vitamin D-deficient symptomatic patients, Vitamin D supplementation was found, using phosphorus-31 magnetic resonance spectroscopy, to augment muscle mitochondrial maximal oxidative phosphorylation after exercise and improved symptoms of fatigue (260). Treatment of skeletal muscle with vitamin D is associated with a change in expression of ~83 nuclear mRNAs encoding proteins known to localize in mitochondria (259).

Hypovitaminosis D is common in critical illness, despite parallel elevations of PTH (249) with one small study reporting a progressive drop in vitamin D levels in the first week of illness (261), while a low 25(OH)D3 status was significantly associated with all-cause and sepsis mortality (236). In early critical illness, vitamin D status is associated with a differential metabolic profile. Glutathione and glutamate metabolism, which play principal roles in redox regulation and immunomodulation, respectively, were significantly upregulated by vitamin D (199). However, evidence of a mortality benefit of vitamin D as monotherapy still remains uncertain (262, 263). A recent large RCT investigated a single dose of 540,000 international units of vitamin D_3_ in critically ill patients with 1,25(OH)D3 levels <20 ng/ml (264). By day 3, the treated group achieved a level of 25-hydroxyvitamin D of 46.9 ± 23.2 ng/ml; measurements of systemic inflammation were not reported. Treatment was not associated with improvement in mortality or secondary variables (264).

### Vitamin C

Ascorbic acid (vitamin C) is a potent water-soluble antioxidant and an enzymatic cofactor that plays a key role in neuro-endocrine and immune homeostatic corrections (265). Most vertebrates can synthesize ascorbic acid from glucose-6-phospate in the liver, with synthesis increasing during stress. In humans and other primates, however, ascorbic acid cannot be synthesized and has to be obtained through the diet. This is the result of a random mutation in the enzyme that catalyzes the final step of ascorbic acid biosynthesis in the common ancestor of the teleost fish some 200 million years ago (266, 267). To date, there is no satisfactory evolutionary explanation for this apparent random loss of ascorbic acid synthetic ability. Individuals from species which have lost the ability to make their own ascorbic acid were not selected against, as long as their diet contained sufficient quantities of vitamin C (266).

Ascorbic acid is actively transported into all cells of the body (except erythrocytes) by the sodium vitamin C transporter-2 (mSVCT2). Ascorbic acid is differentially accumulated by most tissues and body fluids. Studies using radiolabeled ascorbic acid predict that body stores in healthy humans are about 1,500 mg; scurvy is thought to occur when this level falls below 300 mg, with plasma ascorbic acid concentrations <11.3 μM (268). Importantly, the highest concentrations (μM) of ascorbic acid are found in critical organs involved in homeostatic corrections, such as the pituitary gland (2,300–2,800), the adrenals (1,700–2,300), the brain norepinephrine-synthesizing nuclei (800–900), and liver (600–900) (268). This vitamin-sequestering may represent an evolutionary protective or “safety” function.

Ascorbic acid is a key cellular antioxidant. As such, ascorbic acid is an electron donor that directly scavenges for free radicals, and inhibits the generation of new free radicals through its suppressive effects on the NADPH oxidase (NOX) pathway (237). Ascorbic acid also prevents the depletion of other circulatory antioxidants, such as lipid-soluble vitamin E and glutathione, although this is not the case in reverse (200). The anti-oxidant effects of ascorbic acid result in reduced endothelial permeability, improved microvascular and macrovascular function, attenuated cellular apoptosis in pathological states, and improved GR function (237).

Ascorbic acid is maintained at high levels in mature circulating leukocytes (μM amounts in lymphocytes ~3,800; monocytes ~3,100, and neutrophils ~1,400) (268), suggesting an important role in many aspects of the immune response. In leukocytes, ascorbic acid content responds to variations in plasma ascorbate availability (269). Following activation, immune cells undergo dramatic metabolic reprogramming with increased aerobic glycolytic activity and fatty acid oxidation (Warburg effect) under the regulation of hypoxia-inducible factors (HIFs) (270). The result of this change is to rapidly provide ATP and metabolic intermediates for the biosynthesis of immune and inflammatory mediators. Importantly, the hydroxylase enzymes that regulate the actions of the HIFs require ascorbate for optimal activity (269). The immune-enhancing properties of ascorbic acid regulation of HIFs include increased neutrophil and macrophage bacterial killing and phagocytic capacity (269, 271). In addition, ascorbic acid plays an important role in protecting host cells from the excessive oxidative stress caused by infections (265).

**Figure 6:**
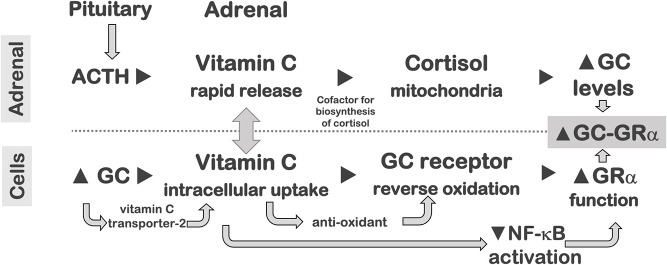
Impact of vitamin C on activated GRα activity. **(Top)** Adrenal: The adrenal and pituitary glands have very high concentrations of ascorbic acid (vitamin C). In response to ACTH release from the corticotrophs of the anterior pituitary gland, the adrenal gland rapidly secretes ascorbic acid, an essential cofactor required for adrenal steroidogenesis in mitochondria, contributing to increased glucocorticoids synthesis. **(Bottom)** Cells: glucocorticoids, in a time- and concentration-dependent manner, increase the expression of mSVCT2, facilitating the uptake of ascorbic acid into the cell. Ascorbic acid reverses oxidation of the GR, restoring cellular glucocorticoid-responsiveness in oxidant conditions. In addition, ascorbic acid inhibits TNFα- and IL-1β-induced activation of NF-κB in a dose-dependent manner by inhibiting phosphorylation and degradation of IκBα. These combined actions result in increased glucocorticoid availability and GC-GRα activation and improved homeostatic corrections.

Ascorbic acid plays a crucial role in HPA axis function (Figure 6). In adrenocortical cells ascorbic acid is sequestered in two pools, one of which can be depleted by ACTH. In response to inflammatory cytokine-mediated ACTH release from the anterior pituitary gland, the adrenal gland rapidly secretes ascorbic acid in amounts that are sufficient to increase, by several fold, plasma ascorbic acid concentrations in the adrenal vein, without increasing systemic levels (268). More than 80 years ago Hans Selye, the pioneer of stress research, reported that the adrenal glands not only contain some of the highest concentrations of ascorbic acid in the human body, but they also employ this vitamin to synthesize cortisol in the adrenal cells (272). Today, *in vitro* and *in vivo* studies have shown that ascorbic acid is an essential cofactor required in both adrenal mitochondrial steroidogenesis and catecholamine biosynthesis (272). The level of ascorbate in the adrenals might affect their capacity to convert cholesterol into pregnenolone, the precursor from which nearly all steroid hormones, including cortisol, are made (273). Additionally, ascorbic acid, as an antioxidant, has a positive impact on GR functions (see section Oxidative Stress and CIRCI).

Oxidative conditions modulate negatively ligand-dependent and independent nuclear import of the GR, affecting GC-GRα DNA binding, and inducible gene expression (225, 229), while a phosphodiester compound of ascorbic acid reverses oxidation of the GR, thereby, restoring the cellular glucocorticoid-responsiveness in oxidant conditions (225). Finally, the cellular uptake of ascorbic acid, mediated by mSVCT2, is downregulated during inflammatory conditions. In a time and concentration-dependent manner, GCs increase the expression of mSVCT2, facilitating the uptake of vitamin C into cells (274), providing the rationale for combination treatment using GCs and ascorbic acid (275). Interestingly, there is a strong inverse correlation between the ability of an animal to endogenously produce vitamin C and the induction of a cortisol response when stressed (276).

In human cell lines and primary endothelial cells (ECV304 and HUVEC), ascorbic acid inhibits TNFα and IL-1β-induced activation of NF-κB, in a dose-dependent manner, by inhibiting phosphorylation and degradation of IκBα (277, 278), independently of its antioxidant properties (277). Preclinical studies show that high-dose vitamin C can prevent or restore microcirculatory flow impairment, reinstate vascular responsiveness to vasoconstrictors, and preserve the endothelial barrier (200). Both ascorbic acid (279) and the GR (see section Endothelium) (130) are essential for endothelial cell homeostasis, and the combination of glucocorticoids with ascorbic acid is superior to either one on its own in protecting vascular endothelium that is critical to allow recovery (280).

Many studies have demonstrated that vitamin C levels are rapidly depleted in critically ill patients, with about 40% of the septic patients having reduced serum levels, similar to those seen at scurvy diagnosis (<11.3 u/mol/l) (281, 282). As intracellular ascorbate concentrations in mononuclear leukocytes and in granulocytes are, respectively, 80 and 25 times higher than in plasma, a high production and turnover of these cells may also contribute to its depletion (200). Low plasma concentrations of vitamin C are associated with more severe organ failure and increased risk of mortality (282). Similar to thiamine, ascorbic acid deficiency syndrome (scurvy) bears a number of similarities to sepsis, including coagulation abnormalities, and breakdown of the endothelial wall (282).

In a phase I safety trial, intravenous ascorbic acid infusion was safe, well-tolerated, and associated with improvement in multiple organ dysfunction and decreased biomarkers of inflammation and endothelial injury (281). Additionally, a small RCT investigating high dose ascorbic acid administration in patients with septic shock reported a reduction in 28-day mortality (283), while a larger trial in patients with sepsis-associated ARDS reported a significant reduction in 28-day all-cause mortality (secondary outcome) (284). The rationale for glucocorticoid treatment in association with high dose ascorbic acid was the subject of recent reviews (237, 267). The promising findings of a recent retrospective study in patients with severe sepsis and septic shock has spurred considerable interest in the subject (275). Randomized data to confirm or refute the observational evidence for the drug combination are needed, and several clinical trials are ongoing or planned in the near future (237).

## Conclusions and Implications for Glucocorticoid Treatment

In critical illness, homeostatic corrections, the culmination of millions of years of evolution, are modulated by the activated GC-GRα and associated with an enormous bioenergetic and metabolic cost. We have reviewed how CIRCI, mitochondrial dysfunction/damage, and hypovitaminosis collectively interact to accelerate an anti-homeostatic active process of natural selection. Importantly, the allostatic overload imposed by homeostatic corrections impacts negatively on both acute and long-term morbidity and mortality, while the bioenergetic and metabolic reserves to support homeostatic corrections are time limited. For these reasons it is prudent to implement early interventions designed to achieve the following: (i) reinforce innate immunity, (ii) inhibit further systemic tissue damage, (iii) limit the metabolic and bioenergetic cacostatic overload imposed during vital organ support, (iv) accelerate disease resolution, and (v) prevent persistent-chronic low-grade systemic inflammation (285). This approach is supported by experimental (286) and clinical studies in patients with septic shock or ARDS (287–290).

The actions of the activated GRα cannot be categorized as merely anti-inflammatory, as it is now clear that insufficient intracellular GRα regulatory action and not relative adrenal insufficiency is the primary driver of CIRCI (17). Therefore, glucocorticoid treatment should not be viewed exclusively as anti-inflammatory or as a hormone replacement for relative adrenal insufficiency. It also is equally relevant that one should recall that full biological resolution lags weeks behind clinical resolution of an acute illness, making the clinical criteria that we frequently employ to guide duration of treatment, an inadequate reference point (291). For these reasons, glucocorticoid treatment, and other co-interventions should be directed at supporting the activated GRα regulatory function throughout all phases of homeostatic corrections, and not limited to the acute phase of organ support.

Randomized studies provide evidence that prolonged glucocorticoid administration is associated with increased GRα number and function and decreased oxidative stress (see sections Glucocorticoid Receptor Alpha in Critical Illness and Mitochondria and HPA-Axis Cross-Talk). Additionally, the activated GRα interdependence with functional mitochondria and three vitamin reserves provides a rationale for co-interventions that include rapid replacement of vitamins B1, C, and D. Recent evidence generated from a retrospective before-after clinical study in patients with severe sepsis has generated momentum for increased research in this field (275), with ongoing confirmatory randomized trials in progress (282).

**Figure 7:**
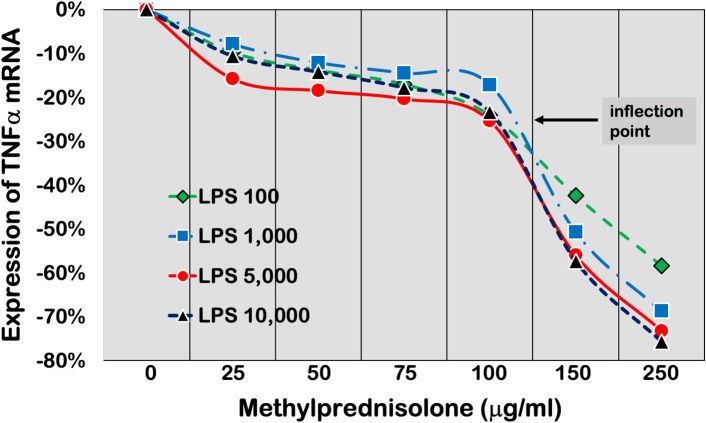
Regression of concentration of methylprednisolone on steady-state mRNA levels of TNF-α in U937 cells primed with graded concentrations of lipopolysaccharide (LPS). In experimental simulation of severe inflammation, human monocytic cells (U937 cells) were activated with graded concentrations of LPS (100 ng/ml, 1.0 μg/ml, 5.0 μg/ml, or 10.0 μg/ml) for 6 h followed by measurement of the expression of inflammatory cytokines [TNF-α (shown), IL-1β, and IL-6]. Graded concentrations of LPS were followed by progressively higher inflammatory cytokine transcription (for TNF-α: 185, 318, 481, and 566, respectively). These cells were then exposed to graded concentrations of methylprednisolone [(μg/ml): 50, 100, 150, 200, 250] for 6 h followed by repeated measurement of inflammatory cytokine expression (see below). The steady state mRNA levels of TNF-α, IL-1β, or IL-6 in LPS-activated cells were reduced by treatment with methylprednisolone in a concentration-dependent manner. The effective dose of methylprednisolone was 175 mg, a value that appeared to be independent of the priming level of LPS and type of mRNA measured (102). Modified with permission from Meduri et al. (102).

Additional co-intervention with critical hormones and mediators involved in homeostatic corrections are also necessary, such as fludrocortisone (174, 292, 293) or vasopressin (294–296) in patients with septic shock. Fludrocortisone is a mineralocorticoid and glucocorticoid receptor agonist that binds to cytoplasmic receptors, activates their translocation into the nucleus and subsequently initiates the transcription of mineralocorticoid- and glucocorticoid-responsive genes (297). The inclusion or exclusion of fludrocortisone, as a co-intervention with hydrocortisone, may partly explain the differences reported in outcomes of some RCTs (see explanation for Figure 7 below) (292, 298). Other potential co-interventions directed at increasing glucocorticoid receptor expression, such as statins (299), melatonin (300), beta-blockers (301), calcium channel blockers (301), or directed at improving mitochondrial function (194, 302, 303) have not been investigated in association with glucocorticoid treatment in acute illness or alone in chronic critical illness.

Present understanding of the activated GC-GRα's role in immunomodulation and disease resolution should be taken into account when re-evaluating how to administer glucocorticoid treatment and in monitoring treatment responses. There are many variables to consider, including the type of GC to be used, timing, dosage, mode of delivery, co-interventions, duration, and tapering. Over the last 40 years, multiple randomized trials investigating GC treatment in critical illness have clearly shown that the design of a treatment protocol has a profound impact on treatment response and outcome (304, 305). The CONSORT (306) and GRADE (307) systems, while useful in evaluating the quality of a randomized trial, unfortunately lack a position on two fundamental elements of a trial design, namely the disease pathophysiology and the pharmacological principles applicable to the investigated drug. Unfortunately, lack of these specific reference points has generated misinterpretation of the literature, fueling a non-sensical controversy that clearly is not serving the patient (308).

Based on this updated pathophysiological understanding, we offer a few observations and make recommendations for future research. Early initiation of treatment, before homeostatic corrections reach exhaustion, is critical and should be directed at approaching maximal saturation of the glucocorticoid receptor (~100 mg of methylprednisolone equivalent) (309). An adequate initial loading bolus is necessary to achieve prompt elevation in plasma levels and to assure higher GRα saturation in the cytoplasm and on the cell membrane for genomic and non-genomic actions, respectively. In human monocytic cells activated with graded concentrations of LPS and then exposed to graded concentrations of methylprednisolone (Figure 7), reduction in inflammatory cytokine transcription was initially modest, then—after reaching an inflection point—followed by a rapid reduction, likely related to achieving maximal drug receptor saturation and adequate time for a measurable effect (102). To achieve optimal results, the initial loading bolus should be followed by an infusion (daily dose over 24 h) to rapidly achieve a steady state. In patients with septic shock, hydrocortisone administered as an infusion vs. an intermittent bolus was associated with more rapid resolution of shock (310), and fewer hyperglycemic episodes (311, 312).

In general, synthetic glucocorticoids are more potent immunoregulators than is cortisol, because they are not subject to endogenous clearance and inhibitors of cortisol activity, including 11βHSD inactivation. Moreover, synthetic glucocorticoids bind the glucocorticoid receptors with higher affinity and remain longer in the cell nucleus, while they bind to mineralocorticoid receptors with lower affinity than do endogenous glucocorticoids, thereby minimizing mineralocorticoid-related side effects (313). Pharmacokinetics and pharmacodynamics of systemically administered glucocorticoids are reviewed in reference (314). Hydrocortisone and methylprednisolone are the two glucocorticoids most often investigated in critical care RCTs (315). In the past, different exogenous glucocorticoids were thought to be qualitatively indistinguishable from each other because they act via the same glucocorticoid receptor, however, qualitative differences have been recently discovered, and one glucocorticoid cannot be simply replaced by another (314). While hydrocortisone was initially chosen as the drug of choice for adrenal replacement, methylprednisolone may actually offer unique advantages over hydrocortisone as follows: (i) greater affinity for the glucocorticoid receptor (316); (ii) higher penetration in lung tissue (important for ARDS or pneumonia), and with longer residence time (317–319), (iii) higher potency of genomic activity especially NF-κB inhibitory activity (320); and (iv) higher potency of non-genomic activity (321). GRα binding affinity, expressed as relative receptor affinity (RRA), correlates with glucocorticoid potency. The log RRAs for selected glucocorticoids are 0.95, 1.62, and 2.0 for hydrocortisone, methylprednisolone, and dexamethasone, respectively (316). A comparison study between these three types of glucocorticoids is needed.

The suggested mode of administration for septic shock [hydrocortisone <400 mg/day for >3 days (315), or hydrocortisone 50 mg QID for 7 days without tapering] is based in part on an outdated pathophysiological model and a misconception about the risk associated with longer duration of treatment (small) and discontinuation without tapering (high). There is some evidence that a treatment duration of 3–7 days directed at reducing acuity of illness (transient reduction in systemic inflammation) might shortchange the full beneficial effects of glucocorticoid therapy (322). The impact of a longer duration of treatment on medium- and long-term mortality, as observed in RTCs of patients with *Pneumocystis jiroveci* pneumonia (323), needs to be investigated.

While glucocorticoids have an important role in supporting homeostatic corrections, this is achieved at the expense of reversible suppression of the HPA axis. In addition, the risk of glucocorticoid treatment-associated adrenal suppression in critically ill patients with dysregulated systemic inflammation is underappreciated. It has been shown that neither the total or the highest dose, nor the duration of glucocorticoid treatment is a significant predictor of HPA axis recovery (324). In the recent “Reduction in the Use of Corticosteroids in Exacerbated COPD trial” that evaluated prednisone 40 mg daily for 5 or 14 days, adrenal suppression was detected at hospital discharge and at 30 days in 38 and 9% of patients, respectively; no differences were detected between 5 or 14 days of glucocorticoid exposure (325). Similarly to the experimental literature (326, 327), critical care RCTs have shown that abrupt glucocorticoid discontinuation after a 3-to-14 days treatment was rapidly followed by a reconstituted inflammatory response with a clinical relapse in approximately one-third of the patients (233, 322, 328, 329), and increased mortality (329). In the LaSRS trial (329), discontinuation of study drug 48 h post-extubation was associated with clinical relapse in one-quarter of methylprednisolone-treated patients. These patients were rapidly returned to mechanical ventilation (MV) without re-institution of study treatment, fared poorly, required additional days of MV and had a 9-fold increased risk of 60-day mortality (*p* = 0.001) in comparison to patients that did not return to MV (330). Gradual tapering is necessary to preserve the disease improvement achieved during glucocorticoid administration, to sustain continuous resolution and restoration of tissue homeostasis, to achieve gradual recovery of the suppressed HPA axis, to forestall disease relapse from reconstituted systemic inflammation, and finally to comply with the Food and Drug Administration package insert warnings (Reference ID: 3032293) (331).

With rapidly expanding knowledge, appreciation of how homeostatic corrections work and how they evolved provides a conceptual framework to understand and appreciate the complex pathobiology of critical illness. We have reviewed emerging literature clearly placing the activated GRα at the center of the homeostatic corrections in the general adaptation to critical illness. Future research directions should include a reassessment of the pharmacological principles that guide glucocorticoid treatment in critical illness and to devise co-interventions to improve cellular responsiveness to glucocorticoids by correcting conditions associated with a reduction in GRα and mitochondrial concentration and function.

## Search Methodology

In addition to the previously used sources (17), we searched the Google Scholar and PubMed databases, employing the following keywords: “glucocorticoid,” “corticosteroid,” “glucocorticoid receptor,” “stress response,” “acute phase response,” “regulation,” “resolution,” “critical illness related corticosteroid insufficiency,” “treatment,” “systemic inflammation,” “dysregulated” systemic inflammation,” “nuclear factor kappa B,” “evolution,” “endothelium,” “mitochondria,” “reactive oxygen species,” ascorbic acid, thiamine, vitamin D, melatonin, “acute,” “long-term,” chronic,” “homeostasis,” “allostasis,” “cacostasis,” and terms related to critical illness, sepsis, septic shock, acute respiratory distress syndrome, “cardiac events.” Manual searching of articles, including reference lists of cited publications, was also performed to avoid omissions.

## Author Contributions

GM conceived and initiated the article and wrote the original draft. GC contributed to the conception and worked on successive drafts.

## Conflict of Interest

The authors declare that the research was conducted in the absence of any commercial or financial relationships that could be construed as a potential conflict of interest.
